# A comprehensive medical Spanish curriculum model: the Vida Medical Spanish Curriculum

**DOI:** 10.1186/s12909-023-04473-0

**Published:** 2023-06-30

**Authors:** Kyle E. Chang, Jennifer Lewis, Alexandra Lopez Vera

**Affiliations:** School of Medicine, California University of Science and Medicine, 1501 Violet Street, Colton, CA 92324 USA

**Keywords:** Medical Spanish, Medical Spanish curriculum, Vida, Hispanic patient population, Health disparities, Limited english proficiency

## Abstract

**Introduction:**

Racial and language disparities in the United States healthcare system have long undermined the quality of care provided to minority patients. With the projected growth of the Hispanic population, there is an urgent need for medical schools to integrate high-quality medical Spanish and cultural competency content. We propose a comprehensive medical Spanish curriculum aligned with the preclinical curriculum as a solution to these issues. The primary goal of this study is to demonstrate the effectiveness of a clinically focused, culturally competent medical Spanish program and advocate for its widespread adoption in medical institutions nationwide.

**Methods:**

The study utilized the Kirkpatrick Model to evaluate the success of the medical Spanish curriculum. A total of 111 medical students voluntarily enrolled in the medical Spanish course. Out of these students, 47 completed the final evaluation, which included a Spanish Objective Structured Clinical Examination and a 40-question Multiple-Choice Exam assessing the integration of Spanish language skills and cultural competency. Both assessment methods took place in clinical skills facilities. Descriptive statistics summarized exam results, and two-tailed t-tests compared mean exam scores between students of different proficiency levels.

**Results and discussion:**

Students achieved a mean score of over 80% on all components of the Spanish Objective Structured Clinical Examination and the Multiple-Choice Exam. Survey data suggest that students felt able to communicate in Spanish with patients after completing the course series. The study also provides a model for a medical Spanish curriculum that applies expert-recommended best practices to meet the needs of Hispanic patient populations.

**Limitations and conclusions:**

Students who sat for the OSCE and MCE were self-selected. Baseline data on student perceptions and Spanish competency are not sufficient for making comparisons.

**Supplementary Information:**

The online version contains supplementary material available at 10.1186/s12909-023-04473-0.

## Introduction

The Hispanic population in the United States is projected to reach 30% by 2050 [1]. In San Bernardino County, California, more than 35% of the population identifies as non-native English speakers and Spanish speakers [2]. Yet, continued racial and linguistic disparities and implicit biases in U.S. healthcare systems lead to the persistent inadequate treatment of minority patients [3, 4]. Though patients with limited English proficiency may receive assistance in their native language using an interpreter, this is not preferred when patients need to share sensitive information with their healthcare providers [5].

To answer the need for an increased population of Spanish-speaking and culturally competent physicians, high-quality medical Spanish curricula are needed in the United States. As many as 78% of U.S. medical schools offered some form of medical Spanish program in 2019, yet only 21% of these met the standards of medical Spanish education revised most recently in 2020 by a multidisciplinary expert panel [6–8]. A 2015 national survey offers insights into the limited quality of existing medical Spanish programming: identifying student time constraints, heterogeneity of students’ Spanish proficiencies, and insufficient faculty support as some of the challenges in providing medical Spanish education [9]. Moving forward, successful medical Spanish curriculum at U.S. medical schools must adopt expert-established best practices and directly address commonly encountered challenges.

Throughout this paper, the terms “Hispanic” and “Spanish-speaking patients" have been used. It is paramount to clarify that not all individuals whose first language is Spanish identify themselves as Hispanic. Similarly, not all self-identified Hispanic people are proficient in the Spanish language.

### Program design

**Fig. 1:**
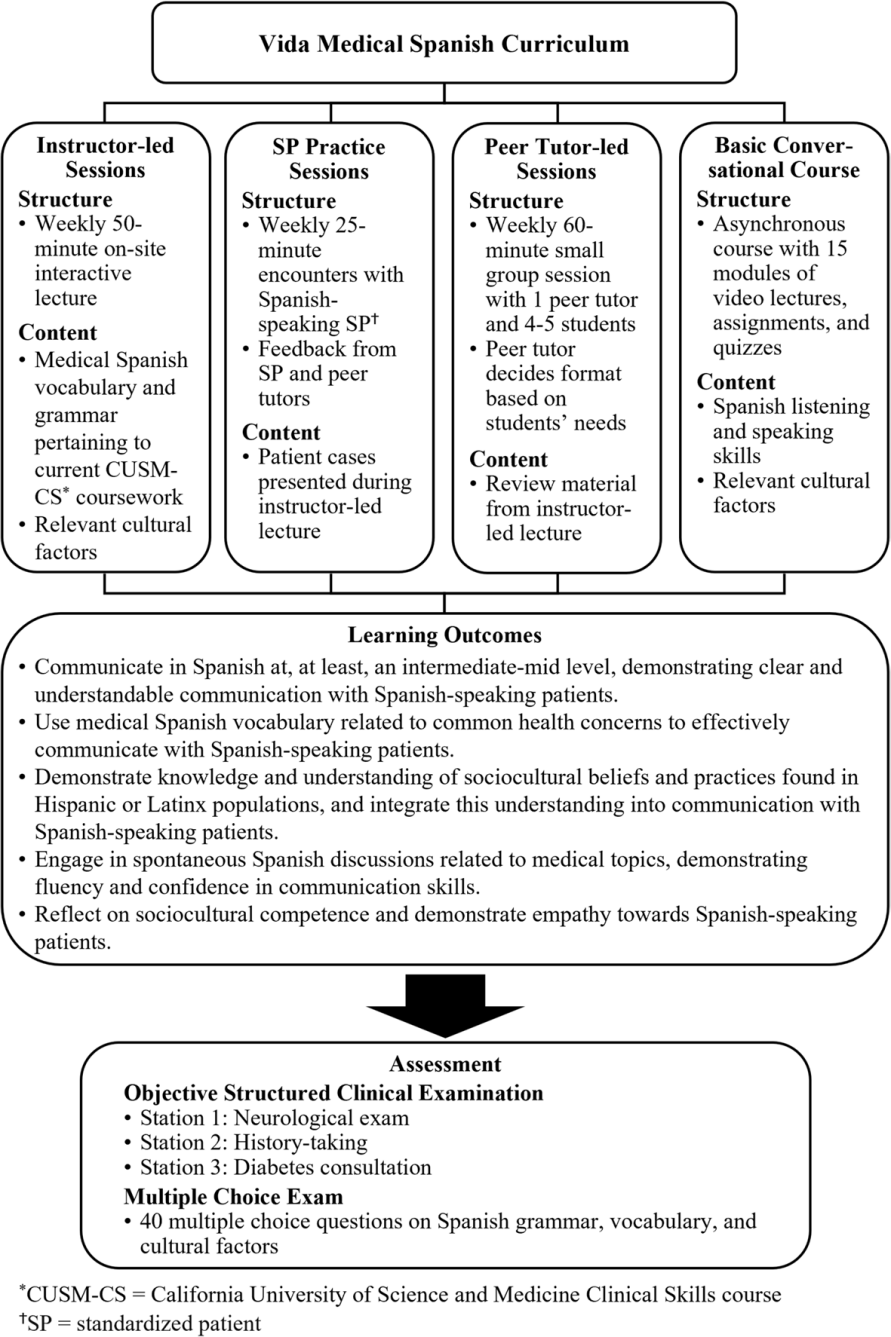
The structure and learning outcomes of the Vida Medical Spanish Curriculum

To meet the demands of the Hispanic population in the Inland Empire region, the California University of Science and Medicine School of Medicine (CUSM-SOM) has made a strong commitment to advancing its Medical Spanish curriculum, known as the Vida course series. The Vida course series has evolved since its inception in 2019 from a model that only included peer-student teaching [10] to the present Vida Medical Spanish Curriculum (VMSC) (Fig. 1). The VMSC is a clinically oriented curriculum structured to equip medical students with the linguistic and cultural competency to provide care to Hispanic patients, consisting of instructor-led lessons, practice with trained standardized patients, peer-led group sessions, and rigorous post-course assessment. The present study focuses on the first cohort of students who completed the Vida course series under the VMSC model from September 2021 to December 2022. These students participated in 18 months of on-site instruction, which was an optional component of the MD curriculum designed for the 2025 graduating class.

The central focus in creating the VMSC was to longitudinally integrate physician–patient communication skills, which students concurrently study in the CUSM-SOM Clinical Skills (CUSM-CS) course series, into a formalized medical Spanish course. We envisioned that an integrated curriculum would have a synergistic effect on each facet of students’ learning: patient cases covered in CUSM-CS prime students with the clinical context for their Spanish studies, while working up the same cases in Spanish during the Vida course series reinforces their clinical knowledge.

To achieve our goals, the VMSC was designed to include weekly instructor-led sessions, interaction with trained Spanish-speaking standardized patients (SPs), and peer tutor-led sessions. Instructor-led sessions focused on improving students’ oral communication skills and were conducted by a faculty instructor with a Ph.D. in second language teaching. In each lesson, the instructor introduced the medical Spanish grammar and vocabulary pertinent to the patient cases that students had studied the week prior in CUSM-CS, as well as relevant cultural factors. Three lesson plan samples are included in Additional file 1. Then, trained Spanish-speaking SPs role-played the same patient cases with the goal of facilitating language practice and improving Spanish communication skills. The incorporation of trained Spanish-speaking SPs into medical Spanish programs has a positive impact on students' ability to communicate in Spanish [11]. Finally, students regularly met in small groups of 4–5 with peer tutors, whose Spanish proficiencies were assessed before being designated as tutors. In peer-led sessions, students discussed and acted out the same patient cases in Spanish using a checklist developed based on the criteria outlined in Bates' Guide to Physical examination and History Taking [12] (Additional file 2). Periodic meetings were held between Vida course series leadership and peer tutors to ensure the equitable delivery of content to all groups, discuss the strengths and weaknesses of each group, and maintain regular oversight.

In addition, the VMSC also included the use of an online basic conversational course, which was designed to help students improve their language skills outside the classroom [13]. This course was made available to students to supplement their language learning and help them practice Spanish at their own pace. The course consisted of interactive online activities such as vocabulary and grammar exercises, audiovisual resources, and practice. Another asynchronous component of VMSC was weekly medical Spanish materials and assessment methods, such as visual presentations, quizzes, and clinical checklists. These materials were created by the Vida director and program leaders to reinforce students’ learning and provide an opportunity to practice what they learn at their own pace.

To provide clarity on the clinically integrated VMSC model, we offer the following written summary of one week in our curriculum: Second-year medical students in the middle of their reproductive health block at CUSM-SOM are studying how to interview a patient who presents with scrotal swelling in CUSM-CS this week. The following week in Vida, these students are taught male reproductive anatomy and relevant history-taking questions such as “¿últimamente, ¿ha levantado objetos pesados?” (*Have you lifted heavy objects recently?*) during their instructor-led session. Then, they practice applying their knowledge in discussions with SPs. Finally, students attend their peer-led small groups to discuss and reinforce their learning.

Finally, it is noteworthy to mention that the VMSC incorporates various interventions aimed at promoting cultural sensitivity and mitigating implicit bias. These interventions include comprehensive training sessions on cultural competence, raising awareness of biases, and providing techniques for effective cross-cultural communication, including the use of gender-affirming language. These measures were designed to enhance students' abilities to navigate diverse patient populations and foster equitable healthcare outcomes.

The purpose of this study is to demonstrate the effectiveness of a clinically focused, culturally competent medical Spanish program and advocate for its widespread adoption in medical institutions nationwide.

## Methods

All students who opted to participate in the Vida course were required to attend weekly small group sessions and complete the post-course assessments, while the instructor-led sessions and practice with standardized patients were strongly encouraged for an optimal learning experience. Spanning 1.5 years, this non-credit optional course aimed to enhance students' ability to effectively communicate with Spanish-speaking patients, thereby strengthening their residency applications and preparing them for their future roles as medical providers. Out of the total enrollment of 111 students, 47 successfully completed the post-course assessments offered in December 2022 and March 2023. With a four-year timeframe to fulfill the requirements of Vida, it is expected that more students will complete the course requirements in December 2023 and beyond. However, the non-mandatory nature of the course introduces limitations, as students have the option to drop it at any point during the four years. The objective was to transition the Vida curriculum into a mandatory course for the M.D. program, starting with the Class of 2028.

All students who chose to participate in the Vida course were obligated to attend weekly small group sessions and fulfill the post-course assessments. Although the instructor-led sessions and practice with standardized patients were labeled as optional, they were highly recommended to ensure an exceptional learning experience.

### Evaluation

The evaluation approach used to evaluate the Vida curriculum and its success included three levels of assessment based on the Kirkpatrick Model described by Smidt et al.: student experience, knowledge, and behavior [14]. Feedback was requested from students at the start, end, and during the course series to ensure that the curriculum met the medical students’ expectations. In addition, the student’s Spanish proficiency was assessed through three different OSCE stations. Finally, a 40-question Multiple-Choice Exam (MCE) and a survey were administered based on topics covered in the curriculum.

A Spanish Objective Structured Clinical Examination (OSCE) was utilized to assess the integration of Spanish language skills in clinical encounters, structured as described by Harden and Gleeson [15] and employing trained Spanish-speaking SPs. This evaluation encompassed three stations: neurological examination, comprehensive history-taking of a patient with endometriosis, and diabetes. The neurological exam was included because it requires diverse Spanish language skills generalizable to many other physical examinations, such as describing physical maneuvers and using formal commands. The comprehensive history-taking station assessed students’ abilities to perform a complete workup of patients while using respectful, sensitive, and inclusive Spanish communication. Finally, the diabetic counseling station was included since diabetes is a disproportionately prevalent and deadly chronic disease among Hispanic patients in the United States [16]. Further, delivering accessible education about disease and culturally competent counseling to Hispanic patients on diet, exercise, medication adherence, and risk factor reduction requires both sophisticated clinical knowledge and sensitive communication skills in Spanish.

The OSCE for second-year examinees was evaluated by third and fourth-year Vida peer tutors using a standardized checklist developed from the criteria outlined in Bates’ Guide to Physical Examination and History-Taking (Additional file 3) [12]. The checklist awarded 0 points if the student failed to perform the action, 1 point if the action was performed with some errors, or 2 points if the action was performed with minimal errors. To ensure consistency among graders, an inter-rater consistency report and recordings of OSCE stations were reviewed by the Vida director. While students had the option to use a copy of the grading checklist during the OSCE as a guide, none used it.

Following the OSCE, a survey and a secure 40-question MCE (Additional file 4) were administered within *ExamSoft*, which is the exam software utilized at CUSM-SOM. The survey collected students’ self-reported proficiency level, confidence in communicating with English-speaking patients, confidence in communicating with Spanish-speaking patients, and confidence in providing care in Spanish to Spanish-speaking patients. The MCE evaluated the student's Spanish vocabulary, grammar, and cultural competency.

### Statistical analysis

We described student cohort demographics and summarized overall exam results using descriptive statistics. Mean exam results were compared between students of different proficiency levels using independent samples grouped with two-tailed *t*-tests. Data compilation and statistical analyses were performed in SPSS (version 28.0.1.0, IBM).

## Results

### Test-taker characteristics

Beginning in September 2021, 111 first-year medical students were enrolled in the optional CUSM-SOM Vida Medical Spanish course series, of whom 55 (50%) were female. The optional nature of the course does introduce certain limitations, as students have the freedom to withdraw from the course at any point during their four years of medical school or choose to take the OSCE or MCE whenever they deem appropriate.

In December 2022, a voluntary cohort of 47 Vida students took the Spanish OSCE and MCE. In this cohort, 26 (55%) students were female, and their Spanish-speaking proficiency levels were evaluated by the instructor at the time of assessment using the standardized levels defined by the American Council on the Teaching of Foreign Languages (ACTFL) [17], with proficiencies ranging from Intermediate-Low to Advanced-Mid (Table 1). Students who did not take their exams in December 2022 either previously withdrew from the course series (*n* = 27, 24%), chose not to take any exams (*n* = 24, 22%), or opted to take their exams at a later date (*n* = 13, 12%).

**Table 1:** Spanish-speaking proficiencies of students who completed Vida Program evaluations in December 2022

ACTFL Spanish Speaking Proficiency	*N* (%)
Intermediate-Low	1 (2)
Intermediate-Mid	17 (36)
Intermediate-High	11 (23)
Advanced-Low	15 (32)
Advanced-Mid	3 (6)

*ACTFL* American Council on the Teaching of Foreign Languages

#### OSCE and MCE exam results

Overall, students achieved a mean score of over 80% on all three components of the Spanish OSCE and on the MCE (Table 2). Students with ACTFL Intermediate speaking proficiency scored similarly to Advanced students in the neurological exam and history-taking OSCE stations, though their history-taking scores only narrowly missed the level of significance (*p* = 0.053). Meanwhile, advanced students scored higher on the diabetes counseling OSCE station. Finally, as expected, students of Advanced proficiency scored higher on the MCE, suggesting that they have higher Spanish linguistic knowledge.

**Table 2:** Mean scores on OSCE and MCE, combined and stratified by ACTFL proficiency

	**Total (*n* = 47)**		**Intermediate**^a^ **(*n* = 29)**		**Advanced**^a^ **(*n* = 18)**		
Exam Component	**Mean (SD)**	**% Mean Score**	**Mean (SD)**	**% Mean Score**	**Mean (SD)**	**% Mean Score**	***P***^†^
**Neurological exam**	111 (15)	85	109 (13)	84	115 (17)	88	0.173
**History-taking**	104 (13)	88	101 (15)	86	108 (8)	92	0.053
**Diabetes counseling**	89 (13)	84	86 (13)	81	94 (11)	89	0.027
**Multiple choice exam**	32 (4)	82	31 (3)	79	34 (4)	87	0.005

^a^ACTFL speaking proficiencies were grouped for comparison (i.e. Intermediate-Low, Intermediate-Mid, and Intermediate-High are combined into ‘Intermediate’ above)

^†^Significant at *P* < 0.05

### Survey

All 47 students who took the OSCE also completed the survey. The survey collected information on students’ perceptions of their Spanish proficiency and communication ability (Table 3). Results suggest that on average, though few participating students felt that they had advanced Spanish proficiency, they felt able to communicate with and provide care in Spanish to patients.

**Table 3:** Results of the survey

Survey Item	N (%)
**What is your current proficiency level in Spanish? (N, %)**	
Advanced	3 (6)
Intermediate	34 (72)
Beginner	10 (21)
**What is your confidence level in providing care in Spanish to Hispanic patients?**	
“High: I feel confident in general terms”	1 (2)
“Moderate: I feel confident in providing basic attention in Spanish, but not in every situation”	39 (83)
“Low: I do not feel able to do it”	7 (15)
**I am confident in my ability to communicate in Spanish with patients.**	
Strongly agree	6 (5)
Agree	33 (30)
Neither agree nor disagree	6 (5)
Disagree	2 (2)
Strongly disagree	0 (0)

## Discussion

To increase the population of Spanish-speaking and culturally competent physicians in the Inland Empire, we developed and delivered a comprehensive medical Spanish curriculum that longitudinally integrates clinical skills and consists of instructor-led sessions, practice with Spanish-speaking standardized patients, peer tutor-led sessions, and an asynchronous basic conversational course. At the end of the curriculum, a voluntary cohort of 47 students completed a rigorous evaluation of three OSCE stations and an MCE. Survey data suggest that these participants felt able to communicate in Spanish with patients after completing the course series.

The VMSC is structured in alignment with multiple published expert recommendations on medical language programming and addresses the common challenges faced across U.S. medical schools in implementing medical Spanish programs [8, 9, 18]. VMSC is a formalized medical Spanish curriculum that focuses on patient-centered communication skills, cultural content, and authentic skills practice. To address the time constraints of medical students’ schedules, we integrated the course contents of the VMSC with the CUSM-CS course to reduce the amount of additional material that our medical Spanish curriculum introduces. Further, the VMSC accommodates heterogeneous student skill levels by providing a basic conversational course and grouping peer-led sessions with students of similar Spanish proficiency. CUSM-SOM has shown its commitment to the Vida course series by providing critical financial support. Institutional investment made it possible to hire a full-time expert faculty at the assistant professor level as the director of the Vida course series and recruit trained native Spanish-speaking standardized patients to facilitate language practice. Finally, for continuous quality improvement, the VMSC utilizes the Kirkpatrick Model [14] to assess the success of its medical Spanish instruction.

Students who successfully completed the Vida curriculum evaluations in the present study ranged from intermediate to advanced Spanish proficiency. Notably, intermediate students did not perform as well as advanced students in all assessment categories. This is in accordance with the objectives of the VMSC, which aims to graduate students with the ability to provide care to Hispanic patients, not specifically with advanced knowledge of medical Spanish. That intermediate and advanced students scored similarly on the neurological exam OSCE station suggests that in performing tasks where physician–patient communication is primarily unilateral, providers of intermediate and advanced Spanish proficiencies may be similarly effective. Other such tasks may include patient intake and other physical exams. On the other hand, as advanced speakers had higher diabetes counseling scores, conversation-based tasks may be better suited for providers of higher proficiencies. To expedite the training of providers with lower proficiency to begin delivering effective care in Spanish, focused linguistic learning tools that teach how to perform physical exams and other similarly unilateral communication tasks in Spanish may be beneficial.

The students’ errors were assessed using the OSCE rubrics, which are provided as additional files, and the oral proficiency of each student was assessed through the analysis of the OSCE observation conducted by the course director, who possesses a doctorate in second language teaching. The course director utilized the ACTFL proficiency guidelines for speaking to evaluate the students' performance. These guidelines encompassed specific criteria such as effective communication, grammatical accuracy, appropriate use of vocabulary and idiomatic expressions, and culturally sensitive expression of meaning. The proficiency level obtained through the assessment serves as a general indicator and is intended for informational purposes only. Students were not explicitly informed of their specific rating since the VMSC followed a pass or no pass grading system. The purpose of the assessment was to provide feedback to students on their overall performance and to identify areas for improvement, rather than assigning specific proficiency levels.

One possible explanation for the difference in performance between the MCE and OSCE could be attributed to the fact that the MCE included grammatical and vocabulary questions that students had to prepare for, creating additional pressure and impacting their performance. In contrast, the OSCE assessments were more extensively practiced during the VMSC, allowing students to become more familiar with the format and requirements. When considering the differences between intermediate and advanced students, advanced proficiency equips them with enhanced linguistic accuracy and fluency. This higher proficiency level empowers students to effectively navigate complex medical scenarios, utilize specialized vocabulary, employ appropriate communication strategies, and adapt their language to meet the specific needs of patients during the diabetes consultation.

In conclusion, the Vida Medical Spanish Curriculum at CUSM-SOM provides a model for a medical Spanish curriculum at the medical school level that applies expert-recommended best practices toward meeting the needs of Hispanic patient populations.

## Limitations & future directions

Our study has limitations. The present sample of students who completed final course evaluations was self-selected due to the elective nature of the Vida course series at CUSM-SOM. Further, the ACTFL Spanish speaking proficiency of student participants was majority Intermediate (61%), with zero Novice proficiency participants. Resultantly, our findings cannot be generalized to students of all proficiency levels. Future implementations of VMSC may consider curricular integration to encourage completion of all course components by students from a wider range of Spanish proficiency levels. This may entail awarding credit for participation or even including Vida in required coursework.

Further, we had insufficient data on students’ Spanish’ proficiency before their participation in Vida. The VMSC includes components to accommodate students of all proficiency levels, ranging from the asynchronous basic conversational course to SP encounters, and provides a comprehensive final course assessment with robust data on student outcomes. However, without sufficient baseline data of student Spanish proficiency and perceptions from our first cohort, we cannot quantify the potential effects that our curriculum has had on outcome measures. Future studies that address this limitation will be especially important in understanding how students of novice proficiency levels perform under the VMSC.

In addition to the recommendations above, future implementations of the VMSC model may consider optimizing the timing of curriculum components. For the student cohort in the present study, the weekly schedule of the CUSM-CS course and Vida course series components was selected based on administrative availability and not by Vida course series leadership. Further, to reduce the burden on administrative resources, it may be beneficial to establish a single, set curriculum evaluation date. In the present study, we offered students the option to take their Vida exams in December 2022 and March 2023 due to overlapping schedules with their CUSM-SOM exams in December 2022.

Finally, the survey results indicate that students underestimate their Spanish abilities. Thus, to improve self-assessment accuracy and enhance students' confidence in their language abilities, potential curricular and programmatic changes can be implemented. These changes involve providing clear proficiency descriptors based on recognized frameworks, facilitating comparative benchmarks with peers of varying proficiency levels, incorporating external evaluation measures, and establishing a system of continuous feedback and reflection. These adjustments could help promote a more objective understanding of language skills, leading to improved self-assessment accuracy and potentially increased confidence among students.

## Supplementary Information

**Additional file 1.**

**Additional file 2.**

**Additional file 3.**

**Additional file 4.**

## Data Availability

The datasets used and/or analyzed during the current study are available from the corresponding author on reasonable request.

## Abbreviations

USM-SOM: California University of Science and Medicine School of Medicine
VMSC: Vida Medical Spanish Curriculum
CUSM-CS: California University of Science and Medicine School of Medicine’s Clinical Skills course series
SP: Standardized patient
MCE: Multiple-choice exam
OSCE: Objective structured clinical examination
ACTFL: American Council on the Teaching of Foreign Languages
