# Supplementary material for: A comprehensive medical Spanish curriculum model: the Vida Medical Spanish Curriculum
Source: BMC Med Educ. 2023 Jun 30;23:488. doi: 10.1186/s12909-023-04473-0 (PMC10311718; doi:10.1186/s12909-023-04473-0)
Supplement: Supplementary file 1 — Additional file 1. [file 12909_2023_4473_MOESM1_ESM.pdf]

# Masculine Genital Problems

**Level:** Second Year Medical Students

**Before the lesson:**

1. Students have to review the target grammar structure and related vocabulary.
2. Students have to read the following article: Espinosa-Hernández, G., Velazquez, E., McPherson, J. L., Fountain, C., Garcia-Carpenter, R., & Lombardi, K. (2020). The role of Latino masculine values in Mexican adolescent sexuality. Cultural Diversity and Ethnic Minority Psychology, 26(4), 520–531. https://doi.org/10.1037/cdp0000328

**Target grammar structure and vocabulary:** Reflexive verbs and the masculine genital system

**Duration:** 75 minutes

**Objectives:**

1. To comprehend the anatomy and physiology of the male reproductive system in Spanish, with an emphasis on the testicles, epididymis, prostate gland, and penis.
2. To recognize and differentiate common disorders and diseases that affect the male reproductive system in Spanish, including but not limited to testicular cancer, epididymitis, and prostatitis.
3. To improve students 'language proficiency in the use of reflexive verbs (such as *inflamarse* or *hincharse*) in Spanish, which are crucial for effectively communicating symptoms and conditions related to the male reproductive system.
4. To gain an understanding of how cultural values, specifically *machismo*, *caballerismo*, are associated with Mexican adolescent sexual behavior and intentions, and how gender, age, and school context may moderate these associations.

**Materials:**

1. PowerPoint presentation
2. Handout with reflexive verbs conjugations and uses
3. Clinical checklist

**Lesson Plan:**

**Warm-up** (5 minutes):

1. Ask "how many different translations of the word "testicles" do you know in Spanish?
2. Allow students to share their answers and write them on the board.
3. Follow up and add more examples from different dialects.

**Presentation** (45 minutes):

## Masculine Genital Problems

1. Begin the lesson by introducing key vocabulary related to the anatomy and physiology of the male reproductive system in Spanish, using visual aids to help students better comprehend the structures and their functions (5 minutes).
2. Have students practice pronouncing and repeating the terms, as well as identifying the Spanish words for different parts of the male reproductive system (5 minutes).
3. Focus on identifying common disorders and diseases that affect the male reproductive system in Spanish. Use a case study similar to the one students saw in clinical skills the previous week to help them apply their understanding of the anatomy and physiology of the male reproductive system in Spanish (5 minutes).
4. Lead a class discussion where students briefly share their analysis of the case study and identify the problem affecting the patient (5 minutes).
5. In pairs, students will practice using verbs such as "inflamarse" and "hincharse" to describe the symptoms and conditions of disorders and diseases affecting the male reproductive system (10 minutes).
6. Recap the key vocabulary and concepts covered in the lesson, and give students a brief opportunity to ask questions or clarify any doubts they may have regarding the target grammar structure (Spanish reflexive verbs) (5 minutes).
7. Discuss the assigned reading and the topics mentioned in it, such as caballerismo and machismo, and ask students for their thoughts on these cultural concepts (8 minutes).
8. Provide students with additional resources such as readings or online exercises to reinforce the objectives covered in the lesson (2 minutes).

**Practice with SPs** (25 minutes):

Students will be divided into groups of three and assigned a standardized patient with a male genital problem. Each student will take turns asking questions. A peer tutor will be assigned to the table to assist with any linguistic issues, and the instructor will also rotate among the tables.

**Neurological Disorders**

**Target grammar structure and vocabulary:** Formal commands

**Duration:** 75 minutes

**Objectives:**

1. To review the basic anatomy and physiology of the central and peripheral nervous systems in Spanish, with an emphasis on the brain and spinal cord.
2. To recognize and differentiate common neurological disorders in Spanish, including but not limited to Alzheimer's disease, Parkinson's disease, and multiple sclerosis.
3. To improve students' language proficiency in the use of formal commands including formal commands with reflexive verbs or direct/indirect object pronouns (such as "levántese" or "descríbame cómo fue su día de ayer") in Spanish, which are crucial for effectively conducting neurological examinations.
4. To gain an understanding of the social and cultural factors that contribute to mental health disparities in the Latino community.

**Materials:**

1. PowerPoint presentation.
2. Handout with formal commands conjugations and uses.
3. Clinical checklist.

**Before the lesson:**

1. Students have to review the target grammar structure of formal commands and related vocabulary.
2. Students have to read the following article: Bucay-Harari L, Page KR, Krawczyk N, Robles YP, Castillo-Salgado C. Mental Health Needs of an Emerging Latino Community. J Behav Health Serv Res. 2020 Jul;47(3):388-398. doi: 10.1007/s11414-020-09688-3. PMID: 32002728; PMCID: PMC7324412.

**Lesson Plan:**

**Warm-up** (5 minutes):

1. Students have to think of a list of common activities or commands that you might give to a patient who displays a neurological problem.
2. Allow students to share their answers in their groups and write them on a paper.
3. Follow up and ask for other examples of formal commands in a medical context.

**Presentation** (45 minutes):

**Neurological Disorders**

1. Begin the lesson by introducing key vocabulary related to the anatomy and physiology of the central and peripheral nervous systems in Spanish, using visual aids to help students better comprehend the structures and their functions (5 minutes).
2. Have students practice pronouncing and repeating the terms, as well as identifying the Spanish words for different parts of the nervous system (5 minutes).
3. Focus on identifying common neurological disorders in Spanish, their symptoms, and treatments. Use a case study similar to the one students saw in clinical skills the previous week to help them apply their understanding of the anatomy and physiology of the nervous system in Spanish (10 minutes).
4. In pairs, students will practice using formal commands to give instructions and recommendations to patients with neurological disorders (10 minutes).
5. Recap the key vocabulary and concepts covered in the lesson, and give students a brief opportunity to ask questions or clarify any doubts they may have regarding the target grammar structure (Spanish formal commands) (5 minutes).
6. Discuss the assigned reading and the topics mentioned in it, such as the mental health needs of Latino individuals (8 minutes).
7. Provide students with additional resources such as readings or online exercises to reinforce the objectives covered in the lesson (2 minutes).

**Practice with SPs** (25 minutes):

Students will be divided into groups of three and assigned a standardized patient with a neurological disorder. Each student will take turns giving instructions and recommendations using formal commands. A peer tutor will be assigned to the table to assist with any linguistic issues, and the instructor will also rotate among the tables.

## History Taking for Patients with Diabetes

**Target grammar structure and vocabulary:** ‘Alguna vez’, ‘pretérito perfecto compuesto’, and medical terms related to diabetes.

**Duration:** 75 minutes

**Objectives:**

1. To develop and apply the Spanish language proficiency required for a clinical history taking, specifically for patients with potential diabetes.
2. To gain an understanding of the relevant medical terms and vocabulary associated with diabetes and its complications in Spanish.
3. To recognize and differentiate the symptoms and complications of diabetes in Spanish.
4. To improve students' understanding of the increasing prevalence of type 2 diabetes among Hispanic populations in the United States..
5. To practice using the target grammar structure of "alguna vez" and the ‘pretérito perfecto compuesto’ together in Spanish in a clinical context.

**Materials:**

1. PowerPoint presentation.
2. Handout with medical terms related to diabetes in Spanish.
3. Clinical checklist.

**Before the lesson:**

1. Students are expected to review the target grammar structure of "alguna vez" and the ‘present perfect’ (“pretérito perfecto compuesto”) in Spanish as well as related vocabulary such as terms related to diabetes in Spanish.
2. Students are also required to read the following article: Aguayo-Mazzucato C, Diaque P, Hernandez S, Rosas S, Kostic A, Caballero AE. Understanding the growing epidemic of type 2 diabetes in the Hispanic population living in the United States. Diabetes Metab Res Rev. 2019 Feb;35(2):e3097. doi: 10.1002/dmrr.3097. Epub 2018 Dec 4. PMID: 30445663; PMCID: PMC6953173.

**Lesson Plan:**

**Warm-up** (5 minutes):

1. Begin the lesson having students divided in groups of 6.
2. Ask the students sit in a circle and ask the first student to say a sentence in Spanish that uses the present perfect tense. For example: "He visitado a mi médico esta semana" (I have visited my doctor this week). The next student in the circle must then repeat the first student's

sentence and add their own sentence using the present perfect tense. For example: "Él me ha recetado un medicamento nuevo" (He has prescribed a new medication for me).

3. Follow up and add more examples from different Spanish-speaking countries.

**Presentation** (45 minutes):

1. Start the lesson by introducing the relevant medical terms and vocabulary associated with diabetes and its complications in Spanish, using visual aids to help students better comprehend the concepts (5 minutes).
2. Have students practice pronouncing and repeating the terms, as well as identifying the Spanish words for different symptoms and complications of diabetes (5 minutes).
3. Provide a brief overview of the history taking process for patients with potential diabetes, including relevant questions and areas of focus (5 minutes).
4. Divide students into small groups and assign each group a role play scenario related to diabetes. Each group will have 5 minutes to prepare and then act out the scenario in Spanish, using the vocabulary and medical terms covered in the lesson (5 minutes).
5. In pairs, students will complete a crossword puzzle related to diabetes vocabulary and medical terms. The puzzle will also include sentences with the target grammar structure of "alguna vez" plus present perfect for students to identify and translate (10 minutes).
6. Students will create a diabetes awareness poster in Spanish, incorporating key vocabulary and concepts covered in the lesson. Students will present their posters to the class and briefly explain their design choices and the message they are trying to convey (5 minutes).
7. Discuss the assigned reading and the topics mentioned in it, such as the diabetes among Hispanics living in the US and the potential strategies to mitigate its impact. Discuss the importance of validating culture (including food) (8 minutes).
8. Provide students with additional resources such as readings or online exercises to reinforce the objectives covered in the lesson (2 minutes).

**Practice with SPs** (25 minutes):

Students will be divided into groups of three and assigned a standardized patient that presents symptoms of diabetes. Each student will take turns asking questions about the patient's history and any relevant symptoms or complications of diabetes. A peer tutor will be assigned to the table to assist with any linguistic issues, and the instructor will also rotate among the tables.
