# Supplementary material for: A comprehensive medical Spanish curriculum model: the Vida Medical Spanish Curriculum
Source: BMC Med Educ. 2023 Jun 30;23:488. doi: 10.1186/s12909-023-04473-0 (PMC10311718; doi:10.1186/s12909-023-04473-0)
Supplement: Supplementary file 2 — Additional file 2. [file 12909_2023_4473_MOESM2_ESM.pdf]

Scrotal Swelling

Setting the Stage

I. Greet patient and introduce self, verify identifying data and pronouns, ensure privacy and comfort, set agenda, ask permission to proceed.

History

I. Elicit chief complaint and SOCRATES
    A. What brings you in today?
        1. ¿Qué le trae a la clínica hoy?
    B. Please tell me more.
        1. Por favor, dígame más.
    C. Where exactly do you feel the discomfort?
        1. ¿Dónde exactamente se siente incómodo?
    D. When did it start?
        1. ¿Cuándo empezó?
    E. Can you describe the discomfort?
        1. ¿Puede describir la incomodidad?
    F. Does it spread anywhere?
        1. ¿Se extiende a otras partes del cuerpo?
    G. Does anything else feel unusual? Do you have any other symptoms?
        1. ¿Se siente algo más anormal? ¿Tiene algún otro síntoma?
    H. Is it constant, or does it come and go?
        1. ¿Es constante, o va y viene?
    I. What makes the discomfort better? Worse?
        1. ¿Qué lo mejora? ¿Qué lo empeora?
    J. On a scale of 1 to 10, how much does it hurt?
        1. En una escala del uno al diez, ¿cuánto le duele?
II. Case-specific questions
    A. How long have you been experiencing this swelling/scrotal fullness?
        1. ¿ Cuánto tiempo lleva sintiendo esta hinchazón/llenura escrotal?
    B. Have you been exercising a lot? Do you lift weights?
        1. ¿Ha hecho mucho ejercicio? ¿Levanta pesas?
    C. Have you lifted heavy objects recently?
        1. ¿Ha levantado objetos pesados recientamente?
    D. Have you had any trauma or accidents affecting this area?
        1. ¿Ha tenido algún trauma o accidente que afecte a esta zona?
    E. Have you had any discharge from the penis?
        1. ¿Ha tenido algún flujo en el pene?
    F. Do you have pain when urinating?
        1. ¿Tiene dolor al orinar?
    G. Have you felt any urgency to go to the bathroom?

1. ¿Ha sentido alguna urgencia por ir al baño?

H. Have you had changes in how frequently you urinate?

   1. ¿Ha tenido cambios en la frecuencia de orinar?

I. Have you noticed any skin changes or discoloration in this area?

   1. ¿Ha notado algún cambio en la piel o decoloración en esta zona?

J. Do you have a fever?

   1. ¿Tiene fiebre?

K. Have you had changes in weight?

   1. ¿Ha tenido cambios de peso?

III. Social History

   A. Are you sexually active?

      1. ¿Está activo/a/e sexualmente?

   B. Does it hurt during intercourse?

      1. ¿Le duelen las relaciones sexuales/ Tiene dolor cuando mantiene relaciones sexuales?

   C. How many sexual partners do you have now?

      1. ¿Cuántas parejas sexuales tiene actualmente?

Focused Vocabulary

I. Penis
   a. El pene

II. Bladder
   a. La vejiga

III. Skin changes or discoloration
   a. Cambios o decoloración de la piel

IV. Circumcision/are you circumcised?
   a. La circuncisión
   b. ¿Está circuncidado?

V. Foreskin
   a. El prepucio

VI. Abnormal curvature
   a. Curvatura anormal

VII. Cryptorchidism/did you have surgery because your testes failed to descend from the abdomen into the scrotum?
   a. Criptorquidia
   b. ¿Se operó porque sus testículos no descendieron del abdomen al escroto?

Grammar Spotlight: Idiomatic Expressions with "Tener"

I. Definition of Idiom according to Merriam-Webster:
   a. An expression in the usage of a language that is peculiar to itself either in having a meaning that cannot be derived from the conjoined meaning of its elements
   b. Ex: "Up in the air" = undecided

II. Idiom: Tengo (tener) _____ años

a. Literal translation: I have ___ years
b. True meaning: I am ___ years old

III. Idiom: Tengo (tener) prisa
a. Literal translation: I have hurry/rush
b. True meaning: I am in a hurry/rush

IV. Idiom: Tienes (tener) razón
a. Literal translation: You have reason
b. True meaning: You are right

V. Idiom: Tiene (tener) ganas de _____
a. Literal translation: He/she/it has desire
b. True meaning: He/she/it feels like ____

VI. For more practice, take this quiz!

Resources

I. 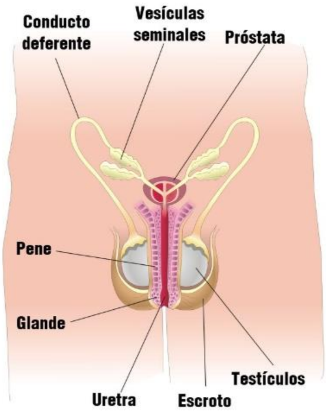

a. Click here for resource

II. Examen fisico del aparato genital masculino

III. Exploracion de genitales masculinos

IV. Beginner: Testiculos inflamados

Scrotal Swelling

Setting the Stage

I. Greet patient and introduce self, verify identifying data and pronouns, ensure privacy and comfort, set agenda, ask permission to proceed.

History

II. Elicit chief complaint and SOCRATES
  a. What brings you in today?
  b. Please tell me more.
  c. Where exactly is the discomfort?
  d. When did it start?
  e. Can you describe the discomfort?
  f. Does it spread anywhere?
  g. Does anything else feel unusual? Do you have any other symptoms?
  h. Is it constant, or does it come and go?
  i. What makes the discomfort better? Worse?
  j. On a scale of 1 to 10, how much does it hurt?

III. Case-specific questions
  a. How long have you been experiencing this swelling/scrotal fullness?
  b. Have you been exercising a lot? Do you lift weights?
  c. Have you lifted heavy objects recently?
  d. Have you had any trauma or accidents affecting this area?
  e. Have you had any discharge from the penis?
  f. Do you have pain when urinating?
  g. Have you felt any urgency to go to the bathroom?
  h. Have you had changes in how frequently you urinate?
  i. Have you noticed any skin changes or discoloration in this area?
  j. Do you have a fever?
  k. Have you had changes in weight?

IV. Social History
  a. Are you sexually active?
  b. Does it hurt during intercourse?
  c. How many sexual partners do you have now?

Upper Extremity Neuro Physical Exam

Setting the Stage

1. Greet patient and introduce self, verify identifying data and pronouns, ensure privacy and comfort, set agenda, ask permission to proceed.

History

I. Elicit chief complaint and SOCRATES
II. Case-specific questions (not included this week)

Physical Exam

I. Inspection of Upper Extremities
   a. I am going to inspect your arms and shoulders.
      i. Voy a inspeccionar los brazos y hombros.
   b. Can you roll up your sleeves?
      i. ¿Puede arremangarse?/ ¿Puede subirse las mangas de la camisa/camiseta?
   c. Lift your arms up in front of you.
      i. Levante los brazos hacia el frente.
   d. Palms down.
      i. Palmas hacia abajo.
   e. Palms up.
      i. Palmas hacia arriba.

   Pronator Drift

   f. Close your eyes and keep your arms up.
      i. Cierre los ojos y mantenga los brazos arriba.
      ii. Cierre los ojos y siga levantando los brazos.
   g. Relax.
      i. Relájese.
      ii. Puede relajarse.

II. Test for Muscle Tone
   a. I am going to move your arm.
      i. Voy a mover el brazo.
   b. Relax.
      i. Relájese.
      ii. Puede relajarse.
   c. And now the other arm.
      i. Ahora al otro brazo.

III. Test for Muscle Strength
   a. I am going to test your muscle strength.
      i. Voy a probar su fuerza muscular.

Shoulders, biceps, triceps, wrist extensors, wrist flexors

b. Lift your arms like this.
    i. Levante los brazos como así.
c. Now, like this.
    i. Ahora, así.
d. Push…
    i. Empuje…
e. Up, down, towards me, towards you.
    i. Arriba, abajo, a mí, a Usted.

Grip

f. Squeeze my fingers.
    i. Apriete mis dedos.

Finger abduction

g. Spread your fingers. Don't let me close them.
    i. Abra los dedos. No me deje cerrarlos.
    ii. Abra los dedos, y que yo no los cierre.

Thumb opposition ("OK" sign)

h. Now, like this.
    i. Ahora, así.
i. Don't let me open your fingers.
    i. No me deje abrir los dedos.
    ii. Que yo no los abra.

IV. Test Deep Tendon Reflexes

a. I am going to use my mallet to test your reflexes.
    i. Voy a usar el martillo para probar los reflejos.
b. Relax.
    i. Relájese.
    ii. Puede relajarse.
c. Can you flex your arm?
    i. ¿Puede flexionar el brazo?
d. And now the other arm.
    i. Ahora al otro brazo.

V. Sensory Testing: Pain

a. I am going to test your sense of touch.
    i. Voy a probar su tacto.
b. Close your eyes and tell me if you feel something sharp or dull.
    i. Cierre los ojos y dígame si siente algo agudo o sordo.
c. Do both sides feel the same?
    i. ¿Se sienten igual los dos lados?/ ¿Siente lo mismo en ambos lados?

VI. Sensory Testing: Light Touch

a. I am going to do the same with a cotton ball.
    i. Voy a hacer lo mismo con una bolita de algodón.
b. Close your eyes and tell me when you feel something.
    i. Cierre los ojos y dígame cuando siente algo.
c. Do both sides feel the same?
    i. ¿Se sienten igual los dos lados?/ ¿Siente lo mismo en ambos lados?

VII. Sensory Testing: Vibration

a. I am going to touch your fingers with my tuning fork.
    i. Voy a tocar los dedos con my diapasón.
b. Close your eyes and tell me when you feel something, and when you stop feeling it.
    i. Cierre los ojos y dígame cuando se sienta algo, y cuando para de sentirlo.
c. Do both sides feel the same?
    i. ¿Se sienten igual los dos lados?/ ¿Siente lo mismo en ambos lados?

VIII. Cortical Function

<u>Stereognosis</u>

a. I am going to put an object in your hand.
    i. Voy a poner un objeto en la mano.
b. Close your eyes and tell me what it is.
    i. Cierre los ojos y dígame qué es.
c. Again.
    i. Otra vez.

<u>Graphesthesia</u>

d. I am going to write a number on your palm.
    i. Voy a escribir un número en la palma.
e. Close your eyes and tell me what it is.
    i. Cierre los ojos y dígame cuál es.
f. Again.
    i. Otra vez.

IX. Tactile Extinction

a. I am going to touch your arms.
    i. Voy a tocar los brazos.
b. Close your eyes and tell me what side I am touching: right, left, or both.
    i. Cierre los ojos y dígame a qué lado estoy tocando: derecho, izquierdo, o ambos.

X. Coordination Testing

<u>Finger-Nose-Finger</u>

a. Touch your nose, then my finger.
    i. Toque la nariz, y luego mi dedo.

b. Keep doing that.
    i. Siga así.

Dysdiadochokinesia

c. Do this.
    i. Haga esto.
d. Keep doing that.
    i. Siga así.

Focused Vocabulary

Verbs

1. Sentirse: To feel (a physical sensation).
2. Levantar: To lift

Adjectives

1. Igual: Same, equal.
2. Agudo, afilado: Sharp
3. Sordo: Dull, deaf

Nouns

1. La diapasón: Tuning fork
2. La bolita de algodón: Cotton ball
3. El brazo: Arm
4. La palma: Palm
5. El dedo: Finger

Phrases

1. No me deje (+ infinitive verb): Don't let me (+ verb)
2. Como así: Like this

Grammar Spotlight: Negative Commands

The neuro strength tests require patients to resist your motions. One common way to prompt patients in English is, "Don't let me (push your arm down)." In Spanish, this requires the use of the negative command, which looks just like the Ud. command form but with a "no" in front of it.

*Don't forget that if you add "que" after the negative command, you will need to use the subjunctive tense thereafter.

**Don't let me** open your fingers.	**No me deje** abrir los dedos.<br>***No deje que yo** abra los dedos. (dejar, Ud.)

**Don’t open** your eyes.	**No abra** los ojos. (abrir, Ud.)
**Don’t forget** to breathe.	**No te olvides** de respirar (olvidarse de, tú)

Resources

Patient fact sheet in Spanish: Examen neurológico (ucsd.edu)

Physical exam guide in Spanish (Advanced): Examen físico neurológico normal (semiologiaclinica.com)

Video (English with Spanish subtitles) – Cómo realizar un examen neurológico en 4 minutos

Video (Demo with Explanations in Spanish) – Exploración física básica del sistema nervioso. Parte 2 - YouTube, Exploración física básica del sistema nervioso. Parte 3 - YouTube

Video – Habilidades prácticas – Exploración neurológica

Upper Extremity Neuro Physical Exam

Setting the Stage

I. Greet patient and introduce self, verify identifying data and pronouns, ensure privacy and comfort, set agenda, ask permission to proceed.

History

I. Elicit chief complaint and SOCRATES
II. Case-specific questions (not included this week)

Physical Exam

I. Inspection of Upper Extremities
   a. I am going to inspect your arms and shoulders.
   e. Can you roll up your sleeves?
   b. Lift your arms up in front of you.
   c. Relax.
   d. Palms down.
   e. Palms up.

   Pronator Drift

   f. Close your eyes and keep your arms up.

II. Test for Muscle Tone
   a. I am going to move your arm.
   b. Relax.
   c. And now the other arm.

III. Test for Muscle Strength
   a. I am going to test your muscle strength.

   Shoulders, biceps, triceps, wrist extensors, wrist flexors

   b. Lift your arms like this.
   c. Now, like this.
   d. Push…
   e. Up, down, towards me, towards you.

   Grip

   f. Squeeze my fingers.

   Finger abduction

   g. Spread your fingers. Don't let me close them.

   Thumb opposition ("OK" sign)

   h. Now, like this.
   i. Don't let me open your fingers.

IV. Test Deep Tendon Reflexes
   a. I am going to use my mallet to test your reflexes.

b. Can you flex your arm?
c. And now the other arm.

V. Sensory Testing: Pain
a. I am going to test your sense of touch.
b. Close your eyes and tell me if you feel something sharp or dull.
c. Do both sides feel the same?

VI. Sensory Testing: Light Touch
a. I am going to do the same with a cotton ball.
b. Close your eyes and tell me when you feel something.
c. Do both sides feel the same?

VII. Sensory Testing: Vibration
a. I am going to touch your fingers with my tuning fork.
f. Close your eyes and tell me when you feel something, and when you stop feeling it.
b. Do both sides feel the same?

VIII. Cortical Function

Stereognosis
a. I am going to put an object in your hand.
b. Close your eyes and tell me what it is.
c. Again.

Graphesthesia
d. I am going to write a number on your palm.
e. Close your eyes and tell me what it is.
f. Again.

IX. Tactile Extinction
a. I am going to touch your arms.
b. Close your eyes and tell me what side I am touching: right, left, or both.

X. Coordination Testing

Finger-Nose-Finger
a. Touch your nose, then my finger.
b. Keep doing that.

Dysdiadochokinesia
c. Do this.
d. Keep doing that.

Consejería sobre la diabetes

Setting the Stage

I. Greet patient and introduce self, verify identifying data and pronouns, ensure privacy and comfort, set agenda, ask permission to proceed.

Counseling

1. Diabetes
    a. Can you tell me what you understand about diabetes.
        i. ¿Puede decirme qué entiende sobre la diabetes?
    b. Diabetes is when our body is unable to control sugar levels.
        i. La diabetes ocurre cuando nuestro cuerpo no puede controlar el nivel de azúcar.
    c. Over time, these high sugar levels can cause damage to the nerves and blood vessels.
        i. Con el tiempo, estos niveles altos de azúcar pueden causar daño a los nervios y los vasos sanguíneos.
    d. It is important to control sugar levels to prevent long-term complications, such as damage to the kidneys, heart and eyes.
        i. Es importante controlar los niveles de azúcar para prevenir complicaciones a largo plazo, como daño a los riñones, el corazón y los ojos.
    e. You can do that by limiting your carbohydrate intake. This includes rice, bread, and potatoes.
        i. Puede hacerlo limitando su consumo de carbohidratos. Esto incluye arroz, pan y papas.
    f. Similarly, you can try doing a bit more exercise, like walking or biking.
        i. También, puede intentar hacer un poco más de ejercicio, como caminar o montar en bicicleta.
2. Weight
    a. I would like to talk about your weight. How do you feel about it?
        i. Me gustaría hablar sobre su peso. ¿Cómo se siente al respecto?
    b. BMI is one way to measure body fat based on height and weight.
        i. El índice de masa corporal (IMC) es una forma de medir la grasa corporal según la altura y el peso.
    c. A BMI of 25 to 29.9 is considered overweight.
        i. Un IMC de 25 a 29.9 se considera sobrepeso.
    d. A high BMI can lead to cardiovascular complications, joint problem, sleep disorder and many more issues.
        i. Un IMC alto puede provocar complicaciones cardiovasculares, problemas articulares, problemas con el sueño y muchos otros problemas.
    e. It is important that we maintain a healthy weight to prevent these complications.
        i. Es importante que mantengamos un peso saludable para prevenir estas complicaciones.
    f. Are you interested in making changes?

        i. ¿Está interesado/a en hacer cambios?
    g. I am happy to help create a plan.
        i. Estoy feliz de ayudar a crear un plan.

3. Exercise
    a. Can you tell me a little bit about your physical activity?
        i. ¿Puede decirme un poco sobre su actividad física?
    b. Do you consider yourself physically active or sedentary?
        i. ¿Se considera una persona fisicamente activa o sedentaria?
    c. How many days a week do you work out?
        i. ¿Cuántos días a la semana hace ejercicio?
    d. What makes it harder to exercise regularly?
        i. ¿Qué hace que sea más difícil hacer ejercicio regularmente?
    e. Exercise is good for your physical and mental wellbeing.
        i. El ejercicio es bueno para su bienestar físico y mental.
    f. It can improve your cardiovascular health and reduce the risks associated with diabetes.
        i. Puede mejorar su salud cardiovascular y reducir los riesgos asociados con la diabetes.
    g. It also improves your mood and helps with sleep.
        i. También mejora su estado de ánimo y ayuda a dormir.
    h. It is recommended that we exercise 30 minutes a day for 5 days a week.
        i. Se recomienda 30 minutos de ejecicio al día, 5 días a la semana.
    i. What can we do to help you get there?
        i. ¿Qué podemos hacer para ayudarlo a lograrlo?
    j. Exercise can include a variety of activities, such as walking, swimming, tennis. It is important to find what you enjoy.
        i. El ejercicio puede incluir una variedad de actividades, como caminar, nadar, tenis. Es importante encontrar lo que le gusta.
    k. Let’s start with small goals. Do you think you could try exercising 2 days a week?
        i. Comencemos con metas pequeñas. ¿Cree que podría intentar hacer ejercicio 2 días a la semana?

4. Diet
    a. How is your diet normally?
        i. ¿Cómo es su dieta normalmente?
    b. Do you eat mainly at home or from restaurants?
        i. ¿Come en casa o en restaurantes?
    c. Do you eat only when you are hungry?
        i. ¿Come solo cuando tiene hambre?
    d. It is important to control your carbohydrate intake with diabetes. All carbs are broken down into sugar, which is contributing to the high sugar levels in the blood.
        i. Es importante controlar su consumo de carbohidratos con la diabetes. Todos los carbohidratos se descomponen en azúcar, lo que contribuye a los niveles altos de azúcar en la sangre.
    e. Carbs include food such as rice, milk, sweets, potatoes and sugars.
        i. Los carbohidratos incluyen alimentos como arroz, leche, dulces, papas y azúcares.
    f. Are you interested in making changes to your diet?
        i. ¿Está interesado en hacer cambios en su dieta?

g. There is a diet called the Mediterranean diet, which could be helpful.
    i. Hay una dieta que se llama “dieta mediterránea”que podría ser útil.
h. It focuses on minimally processed foods, so more plant-based options. It includes healthy fats such as olive oils, nuts, fish and poultry.
    i. Se centra en alimentos mínimamente procesados, por lo que hay más opciones basadas en plantas. Incluye grasas saludables como aceite de oliva, nueces, pescado y pollo.
i. Would you be interested in trying it for a bit? We can modify it to meet your needs.
    i. ¿Estaría interesado en probarlo por un tiempo? Podemos modificarlo para satisfacer sus necesidades.

5. Cholesterol
    a. It seems your cholesterol is a bit elevated as well.
        i. Parece que su colesterol también está un poco elevado.
    b. This can contribute to cardiovascular risks so it is important that we control the levels.
        i. Esto puede contribuir a riesgos cardiovasculares, por lo que es importante que controlemos los niveles
    c. Foods with high levels of cholesterol include red meats, fried food and dairy products.
        i. Los alimentos con altos niveles de colesterol incluyen carnes rojas, alimentos fritos y productos lácteos.
    d. If we can improve your diet and exercise, this should control your cholesterol.
        i. Si podemos mejorar su dieta y ejercicio, esto debería controlar su colesterol.
    e. How does that sound?
        i. ¿Cómo suena eso?
6. Foot care
    a. Diabetes can cause loss of sensation in your feet due to nerve damage.
        i. La diabetes puede causar pérdida de sensación en sus pies debido al daño nervioso.
    b. It is important to check the bottom of your feet regularly to ensure there are no cuts or injuries.
        i. Es importante revisar regularmente la planta de sus pies para asegurarse de que no haya cortes o lesiones.
    c. You can just remove your socks and inspect the sole of your foot and toes.
        i. Usted puede simplemente quitarse los calcetines e inspeccionar la planta del pie y los dedos.
    d. It might be helpful to put an alarm in your phone to remind you to check every day or every other day.
        i. Podría ser útil poner una alarma en su teléfono para recordarle que revise todos los días o cada dos días.
    e. Please do call us if you notice any injuries.
        i. Por favor, llámenos si nota alguna lesión.
    f. Could you show me how you would check your feet?
        i. ¿Podría mostrarme cómo revisaría sus pies?
7. Medication
    a. How has the medication been?
        i. ¿Cómo ha sido la medicación?
    b. Have you noticed any side effects?
        i. ¿Ha notado algún efecto secundario?

c. Has it been difficult to remember to take it every day?
    i. ¿Ha sido difícil recordar tomarla todos los días?
d. I understand it is quite difficult to remember. There are a couple options that could help.
    i. Entiendo que es bastante difícil recordar. Hay un par de opciones que podrían ayudar.
e. You could try putting the pills by your toothbrush or coffee machine, so you see it when you use these items daily.
    i. Puede intentar poner las pastillas junto a su cepillo de dientes o su cafetera, así las verá cuando use estos elementos diariamente.
f. There are pill boxes, that have a box for each day so you can remember if you took the pills each day.
    i. Hay cajas de pastillas que tienen una casilla para cada día para que pueda recordar si tomó las pastillas cada día.
g. You can also set an alarm in your phone to remind you every day.
    i. También puede configurar una alarma en su teléfono para que le recuerde todos los días.
h. It is important to take these pills every day. Although you may not be able to feel the difference if you take the pills or not, they are slowly reducing the levels of sugar in your body. It is important to control the levels before complications develop.
    i. Es importante tomar estas pastillas todos los días. Aunque quizás no pueda sentir la diferencia si las toma o no, están reduciendo lentamente los niveles de azúcar en su cuerpo. Es importante controlar los niveles antes de que se desarrollen complicaciones.

8. Summaries
    a. I know this was a lot of information. Would you mind summarizing what we discussed?
        i. Sé que esto fue mucha información. ¿Podría resumir lo que discutimos?
    b. We will meet in a month to discuss your progress. You've got it!
        i. Nos reuniremos en un mes para discutir su progreso. ¡Usted puede hacerlo!

Focused Vocabulary

I. Diabetes
    a. Diabetes
II. Blood sugar/glucose
    a. Azúcar/glucosa en la sangre
III. Insulin
    a. Insulina
IV. Pancreas
    a. Páncreas
V. Gestational diabetes
    a. Diabetes gestacional
VI. Hypoglycemia / Hyperglycemia
    a. Hipoglucemia / Hiperglucemia
VII. Cryptorchidism/did you have surgery because your testes failed to descend from the abdomen into the scrotum?

a. Criptorquidia
b. ¿Se operó porque sus testículos no descendieron del abdomen al escroto?

VIII. Polyuria / Polydipsia / Polyphagia
  a. Poliuria / Polidipsia / Poligafia

IX. Diabetic ketoacidosis
  a. Cetoacidosis diabética

X. Glucose meter
  a. Medidor de glucosa

Grammar Spotlight: Las Preposiciones

1. Prepositions show the relationship between a noun/pronoun and another word in the sentence.
2. Here are a few examples of prepositions:
   a. A
      i. Indicate direction/destination → Me voy a la biblioteca
      ii. Denote recipient of an action → Ella le dio el libro a su hermana
      iii. Specify a point in time → Nos vamos a las cinco y media
   b. De
      i. Indicate possession → Nos vamos en el coche de Miguel
      ii. Denote origin/source → El es de Guatemala
      iii. Indicate the time of day → Es el 6 de la tarde
   c. En
      i. Location/position of a noun → Estoy en la escuela
      ii. Period of time → Nos vemos en el 2024
      iii. Manner in which something is done → Lo hizo en secreto
   d. Con
      i. With a person → Me voy con el
      ii. With an object → Necesito cortar el pollo con un cuchillo
      iii. With a concept/idea → Estoy feliz con esta nota en el examen
3. Try this quiz
4. !

Resources

1. Testimonio de paciente joven con diabetes tipo 1
2. Dos enfermeras simulan una consulta sobre diabetes para explicar como poner la insulina
3. Especialistas hablan sobre el tratamiento para la diabetes
4. Dia mundial de la diabetes

a. 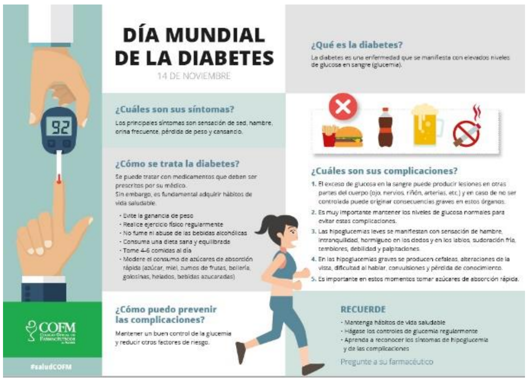

<u>Diabetes Counseling</u>

Setting the Stage

II. Greet patient and introduce self, verify identifying data and pronouns, ensure privacy and comfort, set agenda, ask permission to proceed.

Counseling

9. Diabetes
    a. Can you tell me what you understand about diabetes?
    b. Diabetes is when our body is unable to control sugar levels.
    c. Over time, these high sugar levels can cause damage to the nerves and blood vessels.
    d. It is important to control sugar levels to prevent long-term complications, such as damage to the kidneys, heart and eyes.
    e. You can do that by limiting your carbohydrate intake. This includes rice, bread, and potatoes.
    f. Similarly, you can try doing a bit more exercise, like walking or biking.
10. Weight
    a. I would like to talk about your weight. How do you feel about it?
    b. BMI is one way to measure body fat based on height and weight.
    c. A BMI of 25 to 29.9 is considered overweight.
11. Exercise
    a. Can you tell me a little bit about your physical activity?
    b. Do you consider yourself physically active or sedentary?
    c. How many days a week do you work out?
    d. What makes it harder to exercise regularly?
    e. Exercise is good for your physical and mental wellbeing.
    f. It can improve your cardiovascular health and reduce the risks associated with diabetes.
    g. It also improves your mood and helps with sleep.
    h. It is recommended that we exercise 30 minutes a day for 5 days a week.
    i. What can we do to help you get there?
    j. Exercise can include a variety of activities, such as walking, swimming, tennis. It is important to find what you enjoy.
    k. Let’s start with small goals. Do you think you could try exercising 2 days a week?

12. Diet
    a. How is your diet normally?
    b. Do you eat mainly at home or from restaurants?
    c. What triggers your eating habits?
    d. Do you eat when you are hungry?
    e. It is important to control your carbohydrate intake with diabetes. All carbs are broken down into sugar, which is contributing to the high sugar levels in the blood.
    f. Carbs include food such as rice, milk, sweets, potatoes and sugars.
    g. Are you interested in making changes to your diet?
    h. There is a diet called the Mediterranean diet, which could be helpful.
    i. It focuses on minimally processed foods, so more plant-based options. It includes healthy fats such as olive oils, nuts, fish and poultry.
    j. Would you be interested in trying it for a bit? We can modify it to meet your needs.
13. Cholesterol
    a. It seems your cholesterol is a bit elevated as well.
    b. This can contribute to cardiovascular risks so it is important that we control the levels.
    c. Foods with high levels of cholesterol include red meats, fried food and dairy products.
    d. If we can improve your diet and exercise, this should control your cholesterol.
    e. How does that sound?
14. Foot care
    a. Diabetes can cause loss of sensation in your feet due to nerve damage.
    b. It is important to check the bottom of your feet regularly to ensure there are no cuts or injuries.
    c. You can just remove your socks and inspect the sole of your foot and toes.
    d. It might be helpful to put an alarm in your phone to remind you to check every day or every other day.
    e. Please do call us if you notice any injuries.
    f. Could you show me how you would check your feet?
15. Medication
    a. How has the medication been?
    b. Have you noticed any side effects?
    c. Has it been difficult to remember to take it every day?
    d. I understand it is quite difficult to remember. There are a couple options that could help.
        i. You could try putting the pills by your toothbrush or coffee machine, so you see it when you use these items daily.
        ii. There are pill boxes, that have a box for each day so you can remember if you took the pills each day.
        iii. You can also set an alarm in your phone to remind you every day.
    e. It is important to take these pills every day. Although you may not be able to feel the difference if you take the pills or not, they are slowly reducing the levels of sugar in your body. It is important to control the levels before complications develop.
16. Summaries
    a. I know this was a lot of information. Would you mind summarizing what we discussed?
    b. We reviewed that diabetes is caused by high blood sugar levels and can have long-term consequences.
    c. We will work on controlling the diabetes through increasing exercise habits and improving

diet.

d. Diet includes limiting carbohydrate, cholesterol and sugar intake. This can be accomplished by eating more natural and less-processed food.
e. How does that sound?
f. We will meet in a month to discuss your progress. You’ve got it!
