# Supplementary material for: A comprehensive medical Spanish curriculum model: the Vida Medical Spanish Curriculum
Source: BMC Med Educ. 2023 Jun 30;23:488. doi: 10.1186/s12909-023-04473-0 (PMC10311718; doi:10.1186/s12909-023-04473-0)
Supplement: Supplementary file 3 — Additional file 3. [file 12909_2023_4473_MOESM3_ESM.pdf]

# VIDA Program Final OSCE – Neurologic Exam

Name of Student ___________________________________ Date Completed:

| *Setting the stage of the encounter* | Did not Perform (0) | Performed with Some Errors (1) | Performed with Minimal Errors (2) |
|---|---|---|---|
| 1. Greetings and introduction (introduces self with the name and as a medical student) | ❑ | ❑ | ❑ |
| 2. Disinfects hands prior to shaking hands with patient | ❑ | ❑ | ❑ |
| 3. Verifies name and Age / DOB | ❑ | ❑ | ❑ |
| 4. Illicits preferred name and pronouns | ❑ | ❑ | ❑ |
| 5. Ensures the patients' privacy and comfort | ❑ | ❑ | ❑ |
| 6. Sets the agenda (explains what will happen during the encounter) | ❑ | ❑ | ❑ |
| 7. Asks permission to proceed | ❑ | ❑ | ❑ |

| *Upper Extremity Motor Exam* | Did not Perform (0) | Performed with Some Errors (1) | Performed with Minimal Errors (2) | Used Advanced Commands (+1) |
|---|---|---|---|---|
| 1. Shoulder Abduction | ❑ | ❑ | ❑ | ❑ |
| 2. Elbow Flexion | ❑ | ❑ | ❑ | ❑ |
| 3. Elbow Extension | ❑ | ❑ | ❑ | ❑ |
| 4. Wrist Flexion | ❑ | ❑ | ❑ | ❑ |
| 5. Wrist Extension | ❑ | ❑ | ❑ | ❑ |
| 6. Grip Strength | ❑ | ❑ | ❑ | ❑ |
| 7. Finger Abduction | ❑ | ❑ | ❑ | ❑ |
| 8. Thumb Opposition | ❑ | ❑ | ❑ | ❑ |

| *Upper Extremity Sensory Exam* | Did not Perform (0) | Performed with Some Errors (1) | Performed with Minimal Errors (2) |
|---|---|---|---|
| 1. Pain: ❑ test modality on midline<br>❑ ask patient to detect stimulus<br>❑ ask for bilateral comparison | ❑ | ❑ | ❑ |
| 2. Soft Touch: ❑ test modality on midline<br>❑ ask patient to detect stimulus<br>❑ ask for bilateral comparison | ❑ | ❑ | ❑ |

| *Reflex Exam (one side only)* | Did not Perform (0) | Performed with Some Errors (1) | Performed with Minimal Errors (2) |
|---|---|---|---|
| 1. Triceps | ❑ | ❑ | ❑ |
| 2. Biceps | ❑ | ❑ | ❑ |
| 3. Brachioradialis | ❑ | ❑ | ❑ |
| 4. Knee | ❑ | ❑ | ❑ |
| 5. Achilles | ❑ | ❑ | ❑ |
| 6. Babinski | ❑ | ❑ | ❑ |

# VIDA Program Final OSCE – Neurologic Exam

| ***Cerebellar Exam*** | Did not Perform (0) | Performed with Some Errors (1) | Performed with Minimal Errors (2) | Used Advanced Commands (+1) |
|---|---|---|---|---|
| 1. Finger-to-nose test | ❑ | ❑ | ❑ | ❑ |
| 2. Heel-to-shin test | ❑ | ❑ | ❑ | ❑ |
| 3. Rapid alternating movements | ❑ | ❑ | ❑ | ❑ |
| 4. Gait exam | ❑ | ❑ | ❑ | ❑ |
| 5. Tandem Walking | ❑ | ❑ | ❑ | ❑ |
| 6. Romberg test | ❑ | ❑ | ❑ | ❑ |

| CLOSING THE ENCOUNTER AND PROFESSIONALISM | Did not Perform (0) | Performed with Some Errors (1) | Performed with Minimal Errors (2) |
|---|---|---|---|
| 1. Informs about what happens next | ❑ | ❑ | ❑ |
| 2. Asked the patient if they have any questions or concerns | ❑ | ❑ | ❑ |
| 3. Demonstrated attentiveness via non- verbal body language | ❑ | ❑ | ❑ |
| 4. Used appropriate language and avoided medical jargon | ❑ | ❑ | ❑ |
| 5. Demonstrated professional behavior | ❑ | ❑ | ❑ |

| EVALUATOR AND PATIENT ASSESSMENT OF SPANISH ABILITY | Language is a barrier to encounter (1) | Significant effect on encounter (2) | Moderate effect on encounter (3) | Minor effect on encounter (4) | No negative effect on encounter (5) |
|---|---|---|---|---|---|
| 1. Rate the student's speaking ability (rate, fluidity, pronunciation) | ❑ | ❑ | ❑ | ❑ | ❑ |
| 2. Rate the student's use of medical vocabulary (quantity of words and use of follow-up questions/comments) | ❑ | ❑ | ❑ | ❑ | ❑ |
| 3. Rate the student's ability to use grammar correctly | ❑ | ❑ | ❑ | ❑ | ❑ |
| **4. Rate the student's ability to understand the patient** | ❑ | ❑ | ❑ | ❑ | ❑ |
| 5. (Patient interpretation) Did the patient understand the student? | ❑ | ❑ | ❑ | ❑ | ❑ |
| 6. (Patient interpretation) Did the student demonstrate attentiveness and respect through verbal and non-verbal communication? | ❑ | ❑ | ❑ | ❑ | ❑ |

# VIDA Program Final OSCE – Neurologic Exam

| | Check only one box below |
|---|---|
| Using the scale below, rate the student's ability to interact with a patient: | |
| 0 – Doesn't speak any Spanish | ❑ |
| 1 – Is limited to greetings and goodbyes. | ❑ |
| 2 – Can understand very common medical terminology but wouldn't feel comfortable responding or initiating conversation in Spanish. | ❑ |
| 3 – With difficulty, can speak to patients about very common topics and common anatomy. | ❑ |
| 4 – With relative ease, can speak to patients about very common topics and common anatomy. | ❑ |
| 5 – With difficulty, can speak to patients about more intricate medical and nonmedical terminology. | ❑ |
| 6 – With relative ease, can speak to patients about more intricate medical and nonmedical terminology. | ❑ |
| 7 – With very limited help or while making clinically insignificant mistakes, can conduct an entire patient interaction (history & physical). | ❑ |
| 8 – Can conduct an entire patient interaction without the aid of a translator. | ❑ |
| 9 – I consider this student a fluent Spanish-speaker. | ❑ |

| ***Narrative Feedback** (Comment on the overall performance – communication skills, examination skills, technique)* |
|---|
| |

# VIDA Program Final OSCE – History

Name of Student _______________________________ Date Completed:

| *Setting the stage of the encounter* | Did not Perform (0) | Performed with Some Errors (1) | Performed with Minimal Errors (2) |
|---|---|---|---|
| 1. Greetings and introduction (introduces self with the name and as a medical student) | ❑ | ❑ | ❑ |
| 2. Disinfects hands prior to shaking hands with patient | ❑ | ❑ | ❑ |
| 3. Verifies name and Age / DOB | ❑ | ❑ | ❑ |
| 4. Illicits preferred name and pronouns | ❑ | ❑ | ❑ |
| 5. Ensures the patients' privacy and comfort | ❑ | ❑ | ❑ |
| 6. Sets the agenda (explains what will happen during the encounter) | ❑ | ❑ | ❑ |
| 7. Asks permission to proceed | ❑ | ❑ | ❑ |

| *Patient history* | Did not Perform (0) | Performed with Some Errors (1) | Performed with Minimal Errors (2) |
|---|---|---|---|
| 1. Elicits **chief complaint** (uses open ended question such as "What brings you in" | ❑ | ❑ | ❑ |
| 2. Gives the patient the opportunity to relay the issues in his or her own words (e.g. asks "What more can you tell me about that?" | ❑ | ❑ | ❑ |
| **3. History of presenting illness (HPI).** ❑ Site ❑Onset ❑ Character ❑Radiates ❑ Aggravating ❑ Alleviating ❑ Severity | ❑ | ❑ | ❑ |
| 4. Inquires about **past medical history** ❑ Previous Illness ❑ Surgery ❑ Hospitalization ❑ Trauma / Accidents | ❑ | ❑ | ❑ |
| 5. Inquires about ❑ medications ❑ drug allergies ❑ food allergies ❑ environmental allergies ❑ immunizations | ❑ | ❑ | ❑ |
| 6. Elicits **habits** ❑ alcohol ❑ tobacco ❑ drugs | ❑ | ❑ | ❑ |
| 7. Inquires about **OB/GYN history** ❑menarche ❑ last menstrual period ❑ regularity of period ❑ usual cycle length ❑ vaginal discharge ❑ pregnancy | ❑ | ❑ | ❑ |
| 8. Inquires about **sexual history** ❑ relationship status ❑ sexually active ❑ partners ❑ contraceptives ❑ history of STI ❑ last STI screening | ❑ | ❑ | ❑ |
| 9. Elicits other aspects of **the social history** ❑ diet ❑ exercise ❑ employment ❑ pets ❑ hobbies ❑travel ❑sick contacts ❑ religious preferences | ❑ | ❑ | ❑ |
| 10. Elicits **family history** ❑ parents ❑ siblings ❑ children | ❑ | ❑ | ❑ |
| **11. <u>Review of Systems</u>** ❑ Fever / Weight changes ❑ Neuro ❑ Cardio ❑ Respiratory ❑ GI ❑ Renal ❑ MSK/Skin | ❑ | ❑ | ❑ |
| **12. Summarizes and checks for accuracy of information provided** | ❑ | ❑ | ❑ |

| CLOSING THE ENCOUNTER AND PROFESSIONALISM | Did not Perform (0) | Performed with Some Errors (1) | Performed with Minimal Errors (2) |
|---|---|---|---|
| 1. Informs about what happens next | ❑ | ❑ | ❑ |
| 2. Asked the patient if they have any questions or concerns | ❑ | ❑ | ❑ |

| | | | |
|---|---|---|---|
| 3. Demonstrated attentiveness via non-verbal body language | ❑ | ❑ | ❑ |
| 4. Used appropriate language and avoided medical jargon | ❑ | ❑ | ❑ |
| 5. Demonstrated professional behavior | ❑ | ❑ | ❑ |
| 6. Makes a logical differential diagnosis | ❑ | ❑ | ❑ |

| **Evaluator and Patient Assessment of Spanish Ability** | **Language is a barrier to encounter (1)** | **Significant effect on encounter (2)** | **Moderate effect on encounter (3)** | **Minor effect on encounter (4)** | **No negative effect on encounter (5)** |
|---|---|---|---|---|---|
| 1. Rate the student's speaking ability (rate, fluidity, pronunciation) | ❑ | ❑ | ❑ | ❑ | ❑ |
| 2. Rate the student's use of medical vocabulary (quantity of words and use of follow-up questions/comments) | ❑ | ❑ | ❑ | ❑ | ❑ |
| 3. Rate the student's ability to use grammar correctly | ❑ | ❑ | ❑ | ❑ | ❑ |
| **4. <u>Rate the student's ability to understand the patient</u>** | ❑ | ❑ | ❑ | ❑ | ❑ |
| 5. (Patient interpretation) Did the patient understand the student? | ❑ | ❑ | ❑ | ❑ | ❑ |
| 6. (Patient interpretation) Did the student demonstrate attentiveness and respect through verbal and non-verbal communication? | ❑ | ❑ | ❑ | ❑ | ❑ |

| | **Check only one box below** |
|---|---|
| Using the scale below, rate the student's ability to interact with a patient: | |
| 0 – Doesn't speak any Spanish | ❑ |
| 1 – Is limited to greetings and goodbyes. | ❑ |
| 2 – Can understand very common medical terminology but wouldn't feel comfortable responding or initiating conversation in Spanish. | ❑ |
| 3 – With difficulty, can speak to patients about very common topics and common anatomy. | ❑ |
| 4 – With relative ease, can speak to patients about very common topics and common anatomy. | ❑ |
| 5 – With difficulty, can speak to patients about more intricate medical and nonmedical terminology. | ❑ |
| 6 – With relative ease, can speak to patients about more intricate medical and nonmedical terminology. | ❑ |
| 7 – With very limited help or while making clinically insignificant mistakes, can conduct an entire patient interaction (history & physical). | ❑ |
| 8 – Can conduct an entire patient interaction without limitation or the aid of a translator. | ❑ |
| 9 – I consider this student a fluent Spanish-speaker. | ❑ |

| ***Narrative Feedback (Comment on the overall performance – communication skills, examination skills, technique)*** |
|---|
| |

# VIDA Program Final OSCE – Diabetes Counseling

Name of Student ________________________________ Date Completed:

| ***Setting the stage of the encounter*** | **Did not Perform (0)** | **Performed with Some Errors (1)** | **Performed with Minimal Errors (2)** |
|---|---|---|---|
| 1. Greetings and introduction (introduces self with the name and as a medical student) | ❑ | ❑ | ❑ |
| 2. Disinfects hands prior to shaking hands with patient | ❑ | ❑ | ❑ |
| 3. Verifies name and Age / DOB | ❑ | ❑ | ❑ |
| 4. Illicits preferred name and pronouns | ❑ | ❑ | ❑ |
| 5. Ensures the patients' privacy and comfort | ❑ | ❑ | ❑ |
| 6. Sets the agenda (explains what will happen during the encounter) | ❑ | ❑ | ❑ |
| 7. Asks permission to proceed | ❑ | ❑ | ❑ |

| ***General questions about diabetes*** | **Did not Perform (0)** | **Performed with Some Errors (1)** | **Performed with Minimal Errors (2)** |
|---|---|---|---|
| 1. Verify patient's understanding about diabetes (e.g. knowledge about disease, importance of medication and management) | ❑ | ❑ | ❑ |
| 2. Ask about any existing questions or concerns | ❑ | ❑ | ❑ |

| ***Monitor for complications*** | **Did not Perform (0)** | **Performed with Some Errors (1)** | **Performed with Minimal Errors (2)** |
|---|---|---|---|
| 1. Coronary heart disease ❑ hx of MI, chest pain | ❑ | ❑ | ❑ |
| 2. Cerebrovascular disease ❑ hx of stroke | ❑ | ❑ | ❑ |
| 3. Peripheral artery disease ❑ claudication | ❑ | ❑ | ❑ |
| 4. Nephropathy ❑ urinary frequency, changes | ❑ | ❑ | ❑ |
| 5. Retinopathy ❑ visual impairment/changes | ❑ | ❑ | ❑ |
| 6. Neuropathy ❑ stocking-glove loss of sensation | ❑ | ❑ | ❑ |
| 7. Diabetic foot ❑ unnoticed wounds on feet | ❑ | ❑ | ❑ |
| 8. Summarizes and checks for accuracy of information provided | ❑ | ❑ | ❑ |

# VIDA Program Final OSCE – Diabetes Counseling

| ***Counsel on diabetes management*** | Did not Perform (0) | Performed with Some Errors (1) | Performed with Minimal Errors (2) |
|---|---|---|---|
| 1. Diet ❑ illicit current diet ❑ discuss diabetes-friendly diet (e.g. food type, frequency, portion control, etc) | ❑ | ❑ | ❑ |
| 2. Exercise ❑ illicit current exercise regimen ❑ inquire about patient's preferred method of exercise (e.g. sports, walking, dancing) ❑ agree on plan of action with patient | ❑ | ❑ | ❑ |
| 3. Medications ❑ monitor compliance with medications ❑ discuss strategies to improve compliance | ❑ | ❑ | ❑ |
| 4. Counsel patient to lower risk factors ❑ smoking cessation ❑ blood pressure ❑ weight loss | ❑ | ❑ | ❑ |
| 5. Summary ❑ ask patient to summarize counseling ❑ make additions and corrections | ❑ | ❑ | ❑ |

| ***Closing the encounter and professionalism*** | Did not Perform (0) | Performed with Some Errors (1) | Performed with Minimal Errors (2) |
|---|---|---|---|
| 1. Informs about what happens next | ❑ | ❑ | ❑ |
| 2. Asked the patient if they have any questions or concerns | ❑ | ❑ | ❑ |
| 3. Demonstrated attentiveness via non-verbal body language | ❑ | ❑ | ❑ |
| 4. Used appropriate language and avoided medical jargon | ❑ | ❑ | ❑ |
| 5. Demonstrated professional behavior | ❑ | ❑ | ❑ |

| Evaluator and Patient Assessment of Spanish Ability | Language is a barrier to encounter (1) | Significant effect on encounter (2) | Moderate effect on encounter (3) | Minor effect on encounter (4) | No negative effect on encounter (5) |
|---|---|---|---|---|---|
| 1. Rate the student's speaking ability (rate, fluidity, pronunciation) | ❑ | ❑ | ❑ | ❑ | ❑ |
| 2. Rate the student's use of medical vocabulary (quantity of words and use of follow-up questions/comments) | ❑ | ❑ | ❑ | ❑ | ❑ |
| 3. Rate the student's ability to use grammar correctly | ❑ | ❑ | ❑ | ❑ | ❑ |
| **4. Rate the student's ability to understand the patient** | ❑ | ❑ | ❑ | ❑ | ❑ |
| 5. (Patient interpretation) Did the patient understand the student? | ❑ | ❑ | ❑ | ❑ | ❑ |
| 6. (Patient interpretation) Did the student demonstrate attentiveness and respect through verbal and non-verbal communication? | ❑ | ❑ | ❑ | ❑ | ❑ |

# VIDA Program Final OSCE – Diabetes Counseling

| | Check only one box below |
|---|---|
| Using the scale below, rate the student's ability to interact with a patient: | |
| 0 – Doesn't speak any Spanish | ❑ |
| 1 – Is limited to greetings and goodbyes. | ❑ |
| 2 – Can understand very common medical terminology but wouldn't feel comfortable responding or initiating conversation in Spanish. | ❑ |
| 3 – With difficulty, can speak to patients about very common topics and common anatomy. | ❑ |
| 4 – With relative ease, can speak to patients about very common topics and common anatomy. | ❑ |
| 5 – With difficulty, can speak to patients about more intricate medical and nonmedical terminology. | ❑ |
| 6 – With relative ease, can speak to patients about more intricate medical and nonmedical terminology. | ❑ |
| 7 – With very limited help or while making clinically insignificant mistakes, can conduct an entire patient interaction (history & physical). | ❑ |
| 8 – Can conduct an entire patient interaction without the aid of a translator. | ❑ |
| 9 – I consider this student a fluent Spanish-speaker. | ❑ |

| ***Narrative Feedback (Comment on the overall performance – communication skills, examination skills, technique)*** |
|---|
| |
